# Muscle-derived fibro-adipogenic progenitor cells for production of cultured bovine adipose tissue

**DOI:** 10.1038/s41538-021-00122-2

**Published:** 2022-01-24

**Authors:** Richard G. J. Dohmen, Sophie Hubalek, Johanna Melke, Tobias Messmer, Federica Cantoni, Arianna Mei, Rui Hueber, Rada Mitic, Dirk Remmers, Panagiota Moutsatsou, Mark J. Post, Laura Jackisch, Joshua E. Flack

**Affiliations:** 1 Mosa Meat B.V., Maastricht, The Netherlands; 2 Department of Physiology, Maastricht University, Maastricht, The Netherlands

**Keywords:** Mesenchymal stem cells, Agriculture, Cell biology

## Abstract

Cultured meat is an emergent technology with the potential for significant environmental and animal welfare benefits. Accurate mimicry of traditional meat requires fat tissue; a key contributor to both the flavour and texture of meat. Here, we show that fibro-adipogenic progenitor cells (FAPs) are present in bovine muscle, and are transcriptionally and immunophenotypically distinct from satellite cells. These two cell types can be purified from a single muscle sample using a simple fluorescence-activated cell sorting (FACS) strategy. FAPs demonstrate high levels of adipogenic potential, as measured by gene expression changes and lipid accumulation, and can be proliferated for a large number of population doublings, demonstrating their suitability for a scalable cultured meat production process. Crucially, FAPs reach a mature level of adipogenic differentiation in three-dimensional, edible hydrogels. The resultant tissue accurately mimics traditional beef fat in terms of lipid profile and taste, and FAPs thus represent a promising candidate cell type for the production of cultured fat.

## Introduction

Cultured meat (also known as ‘cell-based’ or ‘cultivated’ meat) is an emerging area of biotechnology that aims to address sustainability issues associated with traditional meat production, by leveraging the proliferation and differentiation capacity of stem cells to produce mature, edible tissues for human consumption in vitro^1–3^. Such technologies could have substantial environmental, sustainability and animal welfare benefits when compared to traditionally reared meat^1,3–5^.

Traditional meat is composed primarily of skeletal muscle tissue, and prototype cultured meat products have thus focussed on the differentiation of stem cells (such as muscle satellite cells) to produce skeletal muscle^6^. Various animal-free scaffolding approaches have been developed to optimise the texture and protein expression of this cultured muscle^7,8^. However, inter- and intramuscular fat tissue (known as ‘marbling’) is also a key component of meat and contributes significantly to its flavour, texture and palatability^9–11^. Animal fat is characterised by a distinct lipid profile^12^ and melting temperature, which is difficult to mimic using plant-based fats. Hence, methods for production of cultured fat are required, though key hurdles remain^13,14^. Current adipose tissue engineering protocols have so far proved unsuitable for production of cultured fat for several reasons, including limitations in adipogenic maturity^15,16^, the use of animal-based scaffolds^17,18^, and a lack of scalability^16,19^.

Several cell types are capable of adipogenic differentiation in vitro, including bone- and fat-derived mesenchymal stem cells (MSCs)^20^, de-differentiated fat (DFAT) cells^21^, embryonic stem cells (ESCs), induced pluripotent stem cells (iPSCs)^2^ and, reportedly, satellite cells (SCs)^22,23^. However, in vivo intramuscular fat is derived predominantly from a population of tissue-resident interstitial stem cells known as fibro-adipogenic progenitor cells, (henceforth referred to as FAPs) which have been identified in numerous species^24–26^, including bovine^27,28^, and which are marked by expression of cell surface receptors such as PDGFRα^28–30^. These FAPs play a poorly understood role in skeletal muscle biology, contributing to muscle regeneration under physiological conditions, but to fibrosis and fatty deposition during pathology^24,25,31–33^. Nevertheless, FAPs offer several potential advantages when it comes to design of cultured meat bioprocesses, including ease of cell purification and accurate recapitulation of traditional intramuscular fat.

Here, we show that FAPs can be easily purified from bovine muscle samples, concurrently with SCs, using a simple FACS-based strategy. These cells can be efficiently expanded for many population doublings, and differentiated to produce cultured fat tissue which closely resembles traditional fat in terms of lipid profile and taste. FAPs therefore represent a viable strategy for the scalable production of cultured fat tissue.

## Results

### FAPs can be purified from bovine muscle

**Fig. 1:**
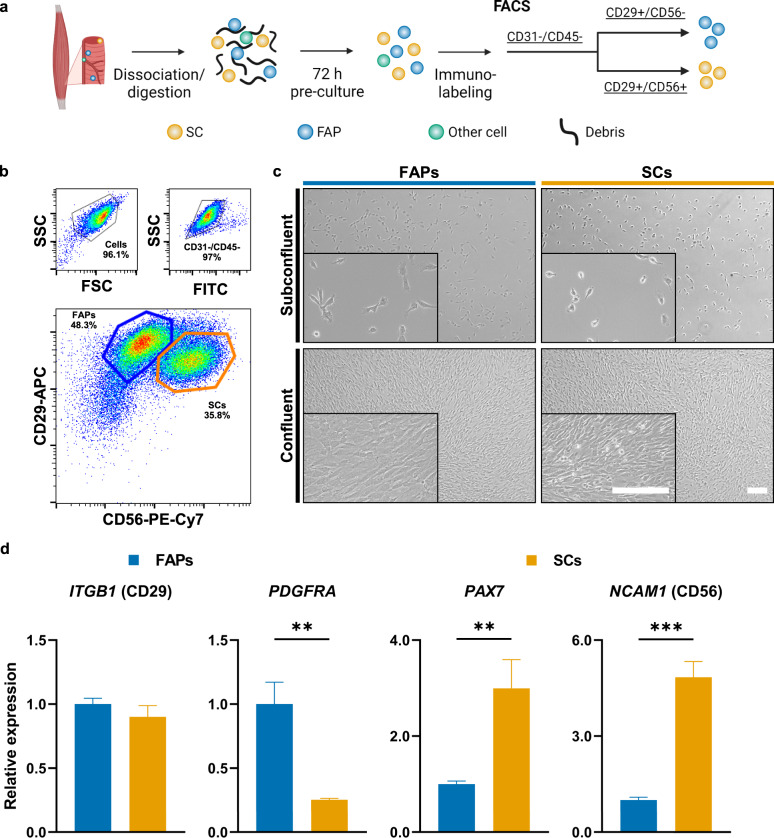
Purification of FAPs from bovine muscle. **a** Overview of workflow for isolation and purification of muscle-derived stem cells. **b** Representative flow cytometry plots of unsorted bovine muscle cells, based on surface expression of CD31/CD45-FITC, CD29-APC, and CD56-PE-Cy7. Coloured gates indicate FACS strategy. Figures denote cells within the respective gates, as a percentage of the parent population. Plots show 50,000 events. **c** Brightfield microscopy images of sorted FAPs and SCs at subconfluence and confluence. Scale bars = 200 µm. **d** Expression of selected stem cell marker genes in FAPs and SCs after sorting, as measured by RT-qPCR. Data was normalised against expression in FAPs. All numerical data is shown as mean ± sd (*n* = 3); *P*-values: *P ≤ 0.05; **P ≤ 0.01; ***P ≤ 0.001; ****P ≤ 0.0001.

Whilst characterising satellite cells (SCs) from bovine muscle samples, we previously observed a substantial population of cells with a CD29+, CD31/45/56- immunophenotype^34^. In order to purify these cells, we employed a FACS strategy similar to that used for the isolation of SCs (Fig. 1a, b)^34^. Sorting this population yielded a culture of mononuclear cells which adhered readily to tissue culture plastic. These cells were larger and distinctly flatter than SCs in vitro (Fig. 1c), and based on previous reports we hypothesised that they might be fibro-adipogenic progenitors (FAPs)^24,25,27,29^. Using RT-qPCR, we found that the CD29+, CD31/45/56- population (which we subsequently refer to as FAPs) showed significantly increased expression of *PDGFRA* (a previously reported FAP marker^27,29^), and significantly reduced expression of *PAX7*^35^ and *NCAM1* (CD56)^34^ when compared to SCs (Fig. 1d). Both of these cell types were isolated with high yields, in the order of 10^5^ cells per gram of skeletal muscle tissue.

### FAPs are transcriptomically distinct from SCs

**Fig. 2:**
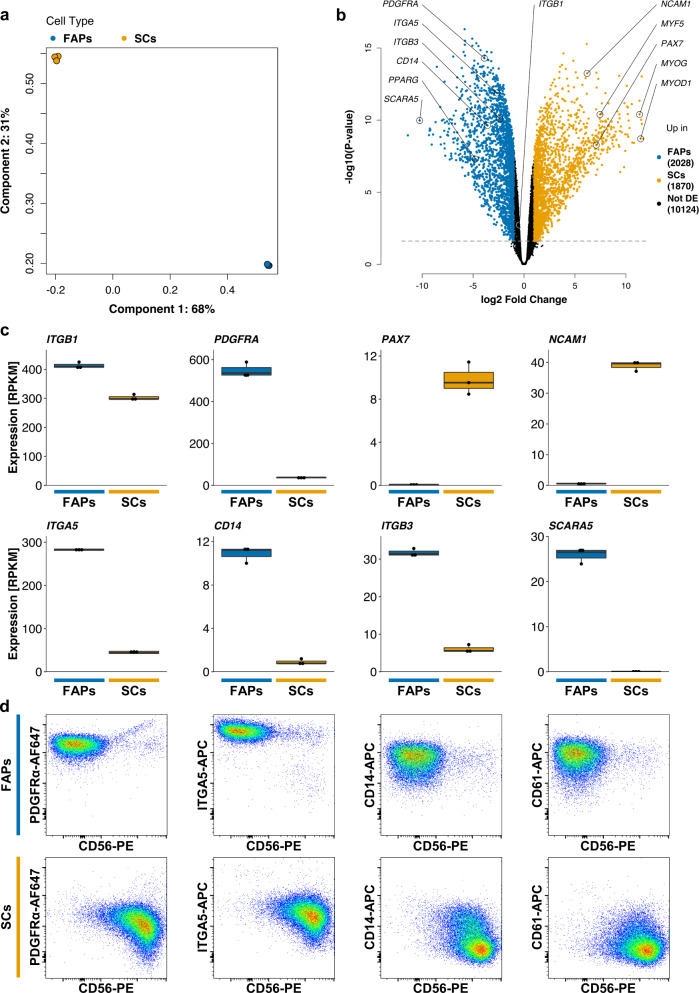
Transcriptomic and immunophenotypic characterisation of FAPs. **a** Principal component analysis of gene expression profiles of FAPs (blue) and SCs (orange) as determined by RNA sequencing, based on the 500 most variably expressed genes (*n* = 3 for each cell type). **b** Volcano plot showing differentially expressed genes between FAPs (blue) and SCs (orange), as determined by RNA sequencing (*n* = 3). Genes corresponding to selected key FAP and SC markers are indicated. Dashed line indicates significance cutoff at a false discovery rate of 0.05. **c** Box plots showing the expression of selected key FAP (blue) and SC (orange boxes) marker genes (*n* = 3), as determined by RNA sequencing. Results are expressed in terms of reads per kilobase million (RPKM). **d** Comparative flow cytometric analysis of FAPs and SCs, based on surface expression of PDGFRα-AF647, ITGA5-APC, CD14-APC, or CD61-APC, each combined with CD56-PE.

Despite recent interest, FAPs remain poorly characterised from both a cellular and physiological perspective^36^. We thus used RNA sequencing to characterise broad transcriptomic differences between FAPs and SCs. Principal component analysis (PCA; based on the 500 most variably expressed genes between samples) demonstrated that samples clustered tightly by cell type (Fig. 2a). Differential expression analysis identified 3898 differentially expressed genes (27.8% of total identified genes; log2-FC > 1, FDR < 0.05) between SCs and FAPs, further demonstrating that these cell types are transcriptomically distinct (Fig. 2b). Previously reported SC markers, including *PAX7*^35^ and *NCAM1*^34^, as well as other myogenic markers, were significantly downregulated in FAPs, as previously observed by RT-qPCR (Figs. 2c; 1d). In contrast, the adipogenic progenitor-related genes *PPARG*^37^ and *SCARA5*^38^, as well as *PDGFRA*, were strongly upregulated in FAPs when compared to SCs (Fig. 2c).

Our transcriptomic comparison additionally identified several cell surface receptors differentially expressed between FAPs and SCs (Fig. 2b, c). We confirmed the selective expression of PDGFRα, ITGA5, CD14, and CD61 at the protein level in sorted FAPs, as compared to SCs, by flow cytometric analysis (Fig. 2d). These data indicate that FAPs and SCs are indeed immunophenotypically distinct, potentially allowing further optimisation of respective purification strategies.

### FAPs can undergo adipogenic differentiation in vitro

**Fig. 3:**
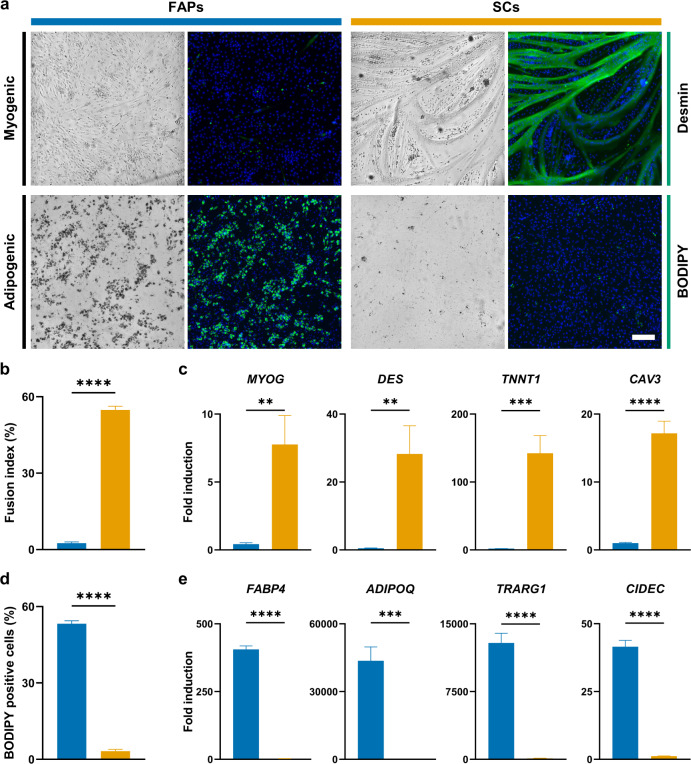
Myogenic and adipogenic differentiation of bovine FAPs and SCs. a Brightfield and immunofluorescence microscopy images of myogenic (top) and adipogenic (bottom panel) differentiation of FAPs and SCs after 96 h (for myogenic) and 14 days (for adipogenic) differentiation in 2D culture vessels, respectively. Green = desmin (myogenic) or BODIPY (adipogenic), blue = Hoechst, scale bar = 200 µm. **b** Quantified fusion indices of myogenic samples in **a**. **c** Expression of muscle-related genes in FAPs and SCs after 96 h of myogenic differentiation, measured by RT-qPCR. Data was normalised against SC control (0 h). **d** Quantification of BODIPY positive cells in adipogenic samples in **a**. **e** Expression of adipogenesis-related genes in FAPs and SCs after 14 days of adipogenic differentiation, measured by RT-qPCR. Data was normalised against FAP control (Day 0). All numerical data is shown as mean ± sd (*n* = 3); *P*-values: *P ≤ 0.05; **P ≤ 0.01; ***P ≤ 0.001; ****P ≤ 0.0001.

To investigate the potential use of FAPs for cultured meat applications, we compared their potential for myogenic and adipogenic differentiation in vitro to that of SCs in 2D assays. After 96 h of serum starvation (traditionally used to induce myogenic differentiation in culture)^39,40^, SCs were observed to form large, desmin-positive myotubes, in contrast to FAPs (Fig. 3a). Myogenic fusion index (the percentage of nuclei present in multinucleated myotubes^41,42^) was significantly reduced in FAPs when compared to SCs (2.4%, SD = 0.5%; 53.8%, SD = 2.3%; Fig. 3b). Expectedly, RT-qPCR analysis confirmed significantly greater upregulation of genes associated with myogenic differentiation in SCs when compared to FAPs (Fig. 3c). Conversely, after 14 days of treatment with an adipogenic differentiation medium (ADM, containing four adipogenic inducer molecules; Supplementary Table 1)^43^, FAPs showed significantly greater accumulation of lipid droplets (53%, SD = 1.1%, compared to 3.2%, SD = 0.7%) than SCs (Fig. 3a, d). Furthermore, FAPs showed potent upregulation of fat-related genes, such as *ADIPOQ* (adiponectin), when compared to SCs (Fig. 3e), indicating that FAPs can differentiate into adipocytes, and suggesting that they warrant further investigation as a starting cell type for cultured fat.

### FAPs can differentiate into mature adipocytes in 3-dimensional hydrogels

**Fig. 4:**
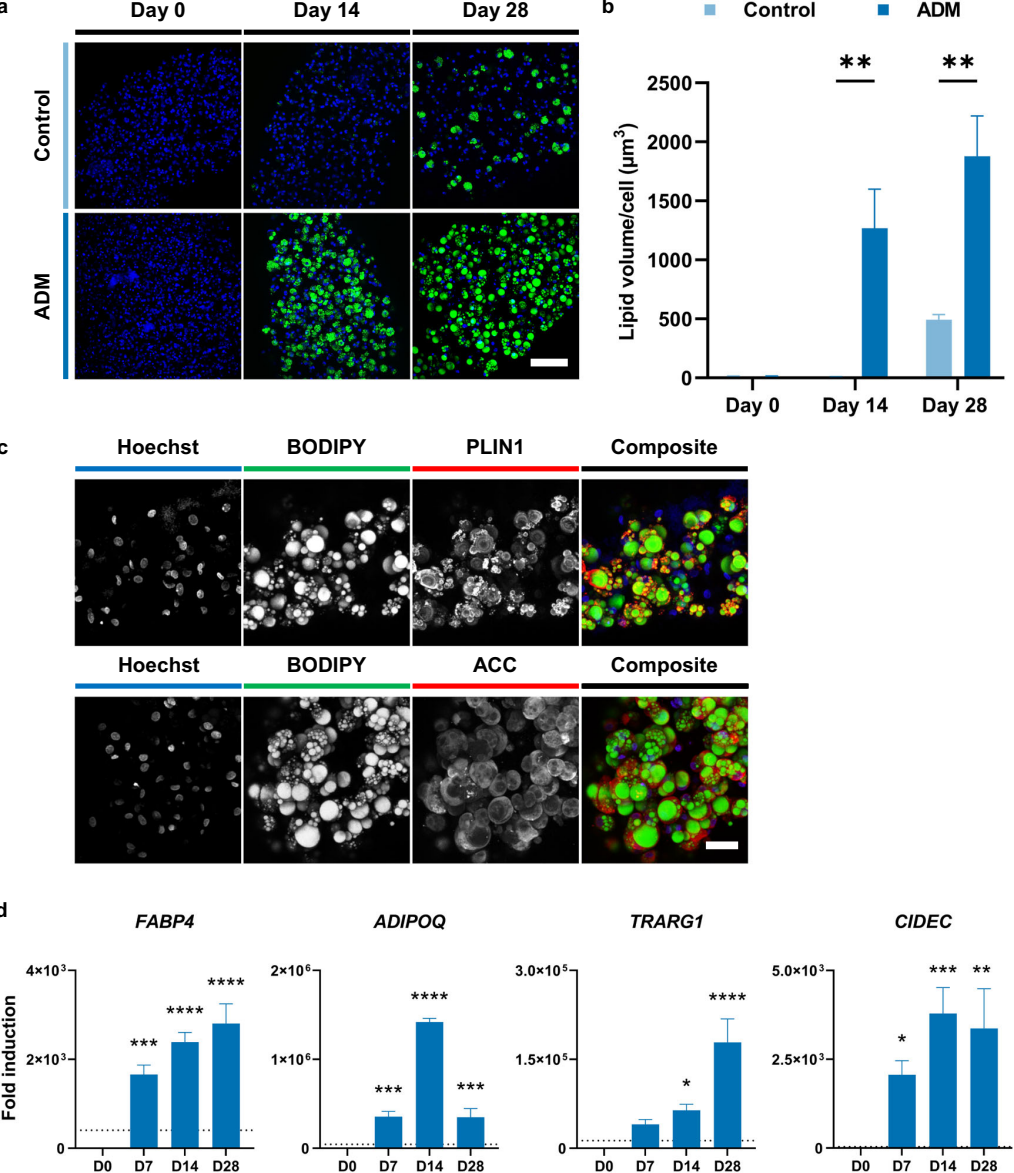
Adipogenic differentiation of FAPs in 3-dimensional hydrogels. a Maximum intensity projection confocal microscopy images of control (top) and ADM (bottom panels) treated FAP microfibres after 0, 14, and 28 days of differentiation. Green = BODIPY, blue = Hoechst, scale bar = 100 µm. **b** Lipid volume per cell, for samples in **a** (quantified by calculating total lipid volume from BODIPY immunofluorescence, divided by number of nuclei, for three independent images). Condition (i.e., control vs ADM) post-hoc significance in a two-way ANOVA is indicated (condition, time, and their interaction are all statistically significant). **c** Maximum intensity projection confocal microscopy images of FAP microfibres after 28 days of differentiation. Green = BODIPY, blue = Hoechst, red = PLIN1 (top) or ACC (bottom panels). Scale bar = 40 μm. **d** Expression of adipogenesis-related genes in FAP microfibres after 0, 7, 14, and 28 days of adipogenic differentiation, measured by RT-qPCR. Data was normalised to the expression on Day 0. Dashed line indicates extent of upregulation after 14 days of 2D differentiation (Fig. 3e). All numerical data is shown as mean ± sd (*n* = 3); *P*-values: *P ≤ 0.05; **P ≤ 0.01; ***P ≤ 0.001; ****P ≤ 0.0001.

Whilst FAPs were able to differentiate into adipocytes in a 2D system, large-scale production of cultured fat requires adipogenesis in the context of 3D tissue biomimetics. FAPs were thus encapsulated in alginate hydrogel and formed into microfibre constructs^15^. Culturing these fibres in ADM gave rise to highly differentiated adipocytes; after 28 days, most of the cells adopted the unilocular lipid droplet morphology characteristic of mature adipocytes (Fig. 4a), in comparison to 2D assays where cells exhibited an accumulation of multiple small fat droplets (Fig. 3a). Quantification of lipid content revealed significantly greater accumulation in ADM (Fig. 4b), although some FAPs differentiated spontaneously in the absence of inducers (control conditions; Fig. 4a). These differentiated microfibres stained strongly for two markers of mature adipocytes: perilipin-1 (PLIN1), at the surface of lipid droplets, and acetyl-CoA carboxylase (ACC), in the cytoplasm (Fig. 4c). Expression of fat-related genes was also highly upregulated upon induction of adipogenic differentiation (Fig. 4d), at significantly higher levels than observed after comparable time in 2D cultures (Fig. 3e). The capacity of lipid accumulation was largely conserved across FAPs derived from different donor animals, although there were significant differences between donors in some cases (Supplementary Fig. 1). These results demonstrate that FAPs are amenable to differentiation in 3D culture, and are thus suitable for production of mature cultured fat tissue.

### FAPs can be expanded effectively in long-term and upscaled cultures

**Fig. 5:**
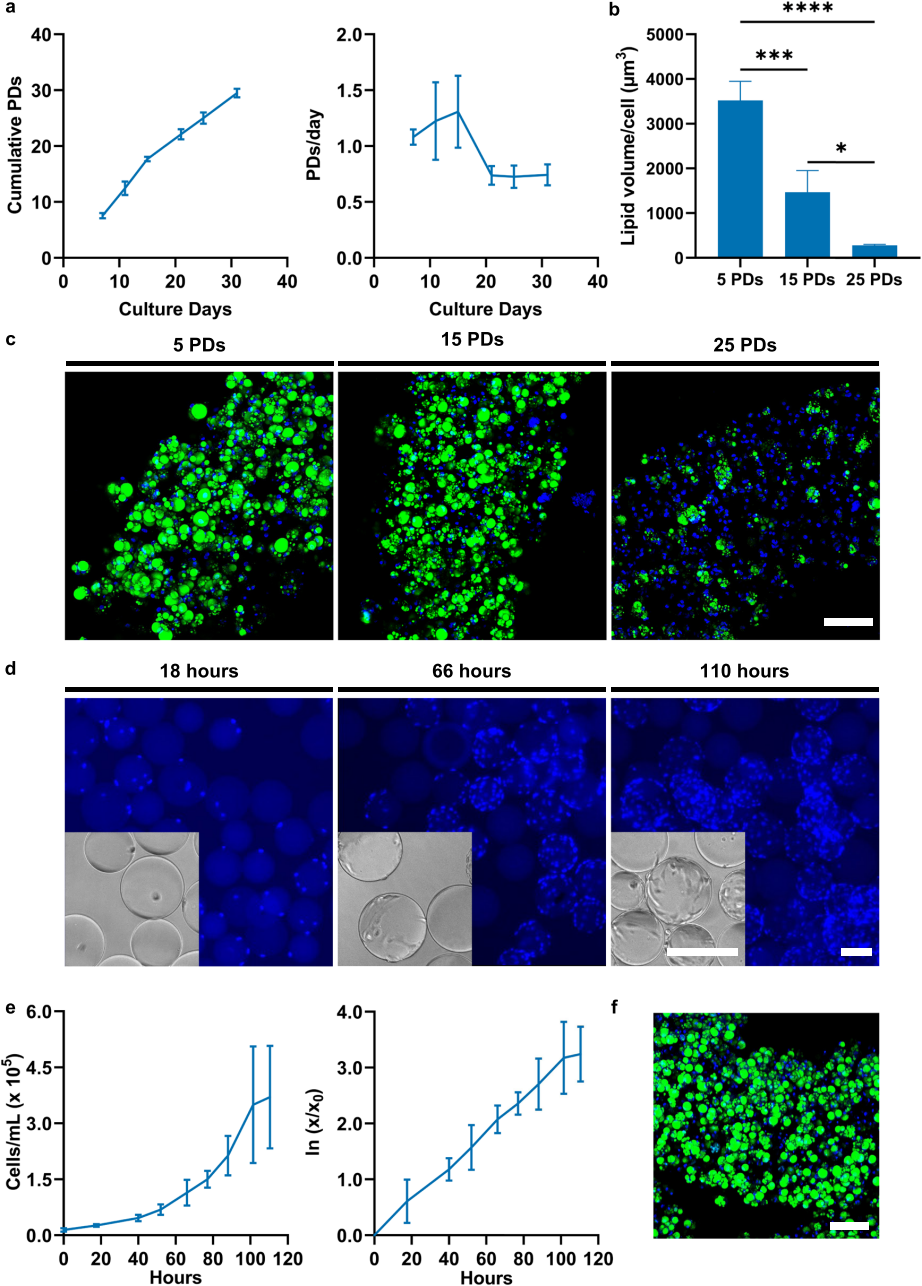
Proliferation of FAPs in long-term and upscaled cultures. **a** Growth curve (left, showing cumulative population doublings) and growth rate (right panel) of FAPs in long-term culture. 95% confidence interval (CI) is indicated (*n* = 3). **b** Lipid volume per cell for 28 day ADM-treated microfibres generated from FAPs that had undergone 5, 15, and 25 population doublings (PDs). Data is shown as mean ± sd (*n* = 4). **c** Representative maximum intensity projection confocal microscopy images of 28 day differentiated microfibres corresponding to the samples quantified in **b**. Green = BODIPY, blue = Hoechst, scale bar = 100 µm. **d** Representative brightfield and immunofluorescence microscopy images of FAPs cultured on Cytodex 1 microcarriers after 18, 66, and 110 h of spinner flask culture. Blue = Hoechst, scale bars = 200 µm. **e** Original (left) and log-transformed (right panel) growth curves for FAPs cultured on Cytodex 1 microcarriers in spinner flasks. 95% CI indicated (*n* = 3). **f** Maximum intensity projection confocal microscopy image of 28 day ADM-treated microfibre created from FAPs proliferated on Cytodex 1 microcarriers. Green = BODIPY, blue = Hoechst, scale bar = 100 µm. *P*-values: *P ≤ 0.05; **P ≤ 0.01; ***P ≤ 0.001; ****P ≤ 0.0001. ADM = adipogenic differentiation medium.

Alongside robust differentiation, efficient expansion of progenitor cells is a requisite for a cultured meat bioprocess. Assessing the long-term proliferative capacity in 2D culture vessels we found that FAPs were able to robustly proliferate for over 30 cumulative population doublings (PDs; Fig. 5a), showing little morphological change over this time (Supplementary Fig. 2a). We also studied the effect of cell ageing on the potential of FAPs to differentiate in adipogenic microfibres. Adipogenesis dropped significantly between early, mid and late passage cells (Fig. 5b), as evidenced by an obvious visual reduction in the proportion of cells accumulating lipid droplets (Fig. 5c). Whilst substantial lipid accumulation was observable at 15 PDs, further work remains to understand and improve this loss of differentiation capacity.

We next analysed the proliferative capacity of FAPs in conditions more suitable for upscaled cell culture. As a proof of concept, FAPs were expanded on Cytodex 1 microcarriers in 30 mL spinner flask cultures. Over a 110 h culture, microcarrier occupancy and confluency visibly increased, with cell density growing 26-fold (Fig. 5d, e). No lag phase was detectable, with an average doubling time of 22.6 h (SD = 2.0 h) during exponential growth (Fig. 5e). Spiking of waste metabolites demonstrated that FAPs were fairly tolerant of lactate and ammonium build-up (to levels of 20 and 3 mM, respectively; Supplementary Fig. 2b), well above the levels of lactate and ammonium we observed in spinner flask cultures (data not shown). Furthermore, after harvesting from microcarriers, FAPs treated with ADM were able to differentiate efficiently in adipogenic microfibres (Fig. 5f). Taken together, these results show that FAPs can be proliferated efficiently, for many PDs, in both planar and microcarrier-based systems suitable for upscaled cell culture.

### Sensory and lipidomic analysis of cultured fat

**Fig. 6:**
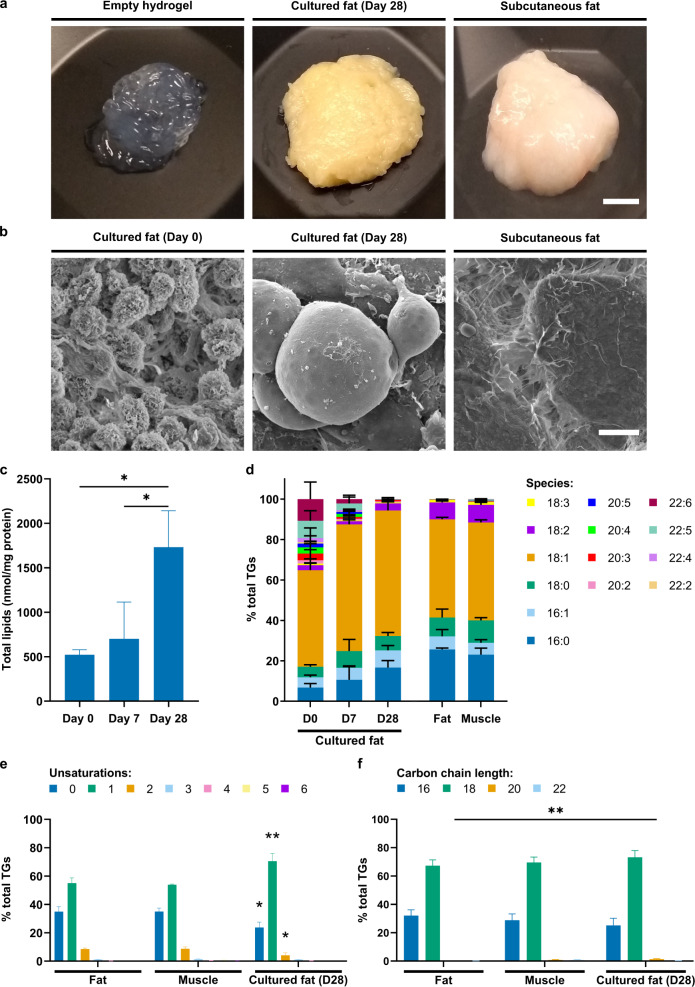
Lipidomic analysis of FAP-derived cultured fat. **a** Macroscopic photographs of empty alginate hydrogel, cultured fat (after 28 days of differentiation), and bovine subcutaneous fat. Scale bar = 5 mm. **b** SEM images of cultured fat after 0 and 28 days of differentiation, and bovine subcutaneous fat. Scale bar = 10 μm. **c** Absolute quantification of total lipid content (normalised to mass of protein) of cultured fat after 0, 7, and 28 days of differentiation. **d** Breakdown of relative percentages of fatty acid species within the triglyceride lipid class in cultured fat samples after 0, 7, and 28 days of differentiation, and bovine subcutaneous fat (‘Fat’) and skeletal muscle (‘Muscle’) control samples. Legend denotes C:D numerical annotation (where C indicates number of carbon atoms, and D number of double bonds, in the acyl chain). **e** Proportion of triglyceride species with acyl chains containing 0 to 6 unsaturations, for three of the samples shown in **d**. Statistically significant differences to both fat and muscle are indicated. **f** Proportion of triglyceride species with acyl chains containing 16, 18, 20, or 22 carbon atoms for the same samples shown in **e**. All numerical data are shown as mean ± sd (*n* = 3). *P*-values: *P ≤ 0.05; **P ≤ 0.01; ***P ≤ 0.001; ****P ≤ 0.0001.

In order for FAP-derived cultured fat to achieve consumer acceptance, it needs to mimic traditional animal fat from a sensory perspective^44^. Cultured fat tissue was produced from FAPs using the alginate microfibre method, on a gram scale, and compared to traditional subcutaneous beef fat (Fig. 6). Macroscopic observation showed clear similarities in appearance and structure between cultured and traditional fat, as compared to the empty alginate hydrogel, although the cultured fat had a more distinct yellow hue, potentially due to accumulation of carotenoid pigments (Fig. 6a). We then performed scanning electron microscopy (SEM) to compare the structures of these samples at the microscale. After 28 days of differentiation, cultured fat cells were considerably larger than at Day 0 (due to lipid accumulation), approaching the size of some cells observed in traditional fat (Fig. 6b). Interestingly, however, subcutaneous fat tissue displayed a branched network of extracellular matrix (ECM) attached to the cells that was not observed at comparable levels in cultured fat. Upon cooking (pan-frying without oil, for 3–5 min), both the traditional and cultured fat samples left oily residues on the pan, though cultured fat also released some water (presumably due to the alginate hydrogel). A panel of three volunteers assessed the sensory properties of the cultured fat tissue, including appearance, aroma, taste, and texture, reporting that the cooked cultured fat had a creamy consistency typical of animal fat, with a discernible ‘beefy’ flavour.

We next performed lipidomic analysis to obtain a quantifiable comparison of cultured fat with traditional bovine subcutaneous fat and muscle tissues. Expectedly, the total lipid content of cultured fat tissue increased significantly during adipogenic differentiation when normalised to protein content (Fig. 6c). During the course of adipogenic differentiation, the triglyceride composition of cultured fat became increasingly similar to that of subcutaneous fat (‘Fat’) and skeletal muscle (‘Muscle’; Fig. 6d), likely reflecting the reported similarity in taste. Interestingly, after 28 days of differentiation, cultured fat contained lower levels of palmitic (C16:0) and linoleic acid (C18:2, an essential fatty acid), and higher levels of oleic acid (C18:1), compared with traditional fat and muscle, which has been reported to correlate positively with palatability (Fig. 6d)^45^. Indeed, the proportion of monounsaturated fatty acids (MUFAs; correlated with desirable flavour attributes^46^) was increased in cultured fat, whilst saturated and polyunsaturated fatty acids were reduced (Fig. 6e). Acyl chain length within the triglyceride class was largely unchanged (Fig. 6f). These results suggest that FAP-derived cultured fat is able to closely mimic traditional beef fat in terms of lipid content and taste.

## Discussion

Cultured meat must accurately mimic both the skeletal muscle and intramuscular fat elements of traditional meat^44^. However, cultured fat has thus far been an often-neglected component in this field^6,23^. In this study, we aimed to address this issue by exploiting the advantageous properties of bovine muscle-derived FAPs, which have not previously received significant attention, for the production of edible cultured fat tissue.

Alongside mature multinuclear muscle fibres, skeletal muscle contains a large number of different cell types^47,48^. Whilst purifying satellite cells by FACS^34^, we had previously observed a large population of CD29+, CD31/45/56- cells. Sorting this population yielded a culture of mononuclear cells possessing distinct morphology from SCs (Fig. 1). Hypothesising that these cells might be FAPs, we confirmed their expression of PDGFRα (CD140a; a tyrosine kinase receptor previously reported to label FAPs in human^26,29^, mouse^24,25^ and bovine^27,28^), as well as other factors previously implicated in adipogenesis, including PPARɣ^37^ and SCARA5^38^ (Fig. 2). These FAPs showed lipid droplet accumulation, as well as strong induction of adipocyte marker genes, upon treatment with a differentiation medium containing a cocktail of adipogenic inducers (Fig. 3). Since FAPs represent the primary source of intramuscular fat deposits in vivo^49,50^, we postulated that cultured fat derived from FAPs might mimic traditional fat tissue more closely than that produced from other cell types, such as subcutaneous fat-derived MSCs. It will be interesting to compare lipid accumulation rates for these bovine cell types, as has been performed for humans^29^. In addition, FAPs are easy to acquire concomitantly with SCs from muscle samples, supporting the development of a commercially viable bioprocess for cultured meat, and are widely conserved across agriculturally relevant species^51^. Our characterisation of FAP cell surface immunophenotypes will help to further optimise sorting strategies for these cell types; for example by combining positive selection for PDGFRα or ITGA5 with known SC markers such as CD56 (Fig. 2)^52^.

Previous studies aiming to produce cultured fat have relied on animal-derived hydrogels or scaffolds^17,18^. Here, we employed alginate-based hydrogels to produce edible cell-laden microfibres^15^, which supported the 3D culture and adipogenesis of FAPs (Fig. 4). The biocompatibility, low cost, and ease of gelation of alginate enable economic scalability^53^, and fibres could feasibly be fabricated as meter-scale constructs^17,54^, cultured in bioreactors to permit efficient nutrient exchange. This hydrogel supported the robust differentiation of FAPs over a four week culture, as evidenced by the presence of unilocular lipid droplets characteristic of white adipocytes (Fig. 3; Fig. 4)^55^. Nevertheless, further understanding and optimisation of cell-biomaterial, cell-cell, and cell-medium interactions will be required to maximise the adipogenic capabilities of FAPs, and to identify a fully-food compatible adipogenic differentiation medium. The tissue origin of FAPs also offers intriguing possibilities for the co-culture of multiple muscle-derived cell types. FAPs reportedly help to promote myofibre repair after muscle injury through the release of paracrine factors, such as IGF-1 and IL-6, that stimulate satellite cell differentiation^25,56^. FAPs also have the capacity to enter a fibroblastic lineage, and the ability to manipulate the differentiation of these cells could permit the production of native extracellular matrix (which we did not observe at considerable levels in this study) to construct more complex tissue mimetics^25,32^.

FAPs, like SCs, are a population of adult stem cells, and as such would be expected to have limited proliferative capacity. However, observed proliferation rates were promising, with FAPs demonstrating robust doubling times even after 20 population doublings (Fig. 5). This may reflect an intrinsic property of the cells, given the propensity for intramuscular fat to increase during aging^9,10^, and is highly advantageous from the perspective of a cultured meat bioprocess^57^. FAPs were also able to proliferate rapidly in microcarrier-based spinner flask cultures at rates similar to those previously observed in 2D culture^58^, whilst retaining their capacity for adipogenic differentiation. Further studies will be required to maximise obtained cell densities, eliminate FBS from proliferation medium formulations, and to fully assess the effects of prolonged proliferative phases in upscaled culture vessels^2^, particularly given the drop in differentiation capacity we observed during long term passaging (Fig. 5). It will be interesting to discern the pathways involved in this loss of stemness, perhaps using transcriptomic approaches. While such studies should allow the useful cellular lifespan to be extended, allowing production on a kilogram scale from single tissue biopsies, frequent biopsying will nonetheless be required, and careful selection of stem cell donors necessary to reduce donor-to-donor variability (which was observed to some extent with respect to differentiation in this study; Supplementary Fig. 1). The biology of adipogenesis changes during aging^59^, whilst differences in fatty acid composition have also been observed between muscles^12,60^, offering intriguing possibilities for donor optimisation.

FAP-derived cultured fat tissue appeared visually similar to traditional animal fat on both a macroscopic and microscopic level, albeit with some colour difference. Lipidomic analysis of mature cultured fat revealed that the triglyceride composition (the most prevalent lipid class, known to be crucial for flavour^45,46^) was similar to that of traditional animal fat tissue (Fig. 6), likely reflecting the close similarity in taste reported in our volunteer testing (although further blind tastings are certainly also required). The majority of the most prevalent fatty acid species have been correlated with palatability of meat, although it is unclear to what extent fatty acid composition per se, compared to the levels of intramuscular fat, contribute to this^60^. Comprehensive understanding of the adipogenic biology of FAPs may in future allow engineering of cultured fat products with specifically altered fatty acid compositions; for example to favour polyunsaturated fatty acids, which have been associated with reduced risk of cardiovascular disease^61^, or to increase levels of essential fatty acids (such as linoleic acid).

Collectively, this work demonstrates that FAPs are a candidate cell type for the production of cultured fat tissue as a novel food ingredient, offering potentially significant advantages with respect to ease of acquisition, mimicry to traditional fat, and co-culture with satellite cells to produce complex tissues. Key challenges remain, including the maintenance of differentiation potential at higher PDs, and the transition to protocols that are fully compatible with mass-market food production. Nevertheless, this represents an important step towards making cultured meat a reality.

## Methods

### Cell isolation

Fresh bovine skeletal muscle and subcutaneous fat samples were obtained from a registered abattoir according to national guidelines on animal tissue handling. Ethical approval was not required for acquisition of muscle and fat samples from commercially slaughtered cattle. Samples were acquired and transported in accordance with Dutch national guidelines on handling of animal material. Mosa Meat B.V. has been granted license to handle Category 3 animal material.

Muscle-derived cells were isolated from the semitendinosus muscle of commercially slaughtered Belgian Blue cattle (both male and female, aged from 1 to 7 years) as previously described^34^. Briefly, muscle was minced and dissociated with collagenase (CLSAFA, Worthington; 1 h, 37 °C). Cell slurries were filtered through a 100 μm cell strainer and incubated in ammonium-chloride-potassium (ACK) erythrocyte lysis buffer (1 min, room temperature (RT)). Cells were resuspended in growth medium (GM; Supplementary Table 1), and filtered through a 40 μm strainer prior to culture.

### Fluorescence-activated cell sorting (FACS)

Prior to FACS, cells were cultured for 72 h on bovine collagen type I (2.5 μg/cm^2^; Sigma-Aldrich, C2124) coated flasks and sorted using antibodies previously described^34^ on a FACSAria II Cell Sorter (BD). CD31-/CD45- cells were sorted into two populations: CD29+/CD56- (FAPs) and CD29+/CD56+ (SCs). Unstained cells were used to define gating parameters, and sorting purities routinely checked by reanalysing the sorted fractions.

### Cell culture

Cells were cultured on collagen-coated flasks in GM at a seeding density of 2 × 10^3^ cells/cm^2^. For myogenic differentiation, cells were plated at 5 × 10^4^ cells/cm^2^ on 0.5% Matrigel in GM for 24 h, and differentiation initiated by switching to myogenic differentiation medium (Supplementary Table 1). For adipogenic differentiation, cells were seeded at 4 × 10^4^ cells/cm^2^ in GM and after 24 h switched to adipogenic differentiation medium (ADM) containing four inducer molecules. After 3 days, medium was exchanged for ADM containing only rosiglitazone and insulin (ADM; Supplementary Table 1). Medium exchanges were performed every three days. Cells treated without inducers served as a control for adipogenic differentiation. Brightfield microscopy images were captured with an EVOS M5000 microscope (Thermo Fisher).

### RT-qPCR

RNA was isolated using the E.Z.N.A MicroElute Total RNA Kit (proliferative and myogenic samples) or E.Z.N.A Total RNA kit II (adipogenic samples; Omega Bio-tek, R6831, or R6934, respectively). RNA was reverse transcribed using the iScript cDNA synthesis kit (Bio-Rad, 1708891) according to the manufacturer’s instructions. RT-qPCR was performed using iQ SYBR Green Supermix (Bio-Rad, 1708880) with primer pairs detailed in Supplementary Table 2. 2^ΔCt^ values for genes of interest were normalised to the average of three housekeeping genes (*B2M*, *RPL19*, and *RPLP0* for myogenic samples; *RPL19*, *RPLP0*, and *UXT* for adipogenic samples).

### RNA sequencing

RNA was harvested from FAP and SC samples using the E.Z.N.A MicroElute Total RNA Kit. Sequencing libraries were prepared using the TruSeq stranded mRNA kit (Illumina), and sequenced on a high-output 75 bp NextSeq 500 (Illumina). STAR aligner 2.7^62^ was used to align single-end reads to the reference genome bosTau9 (ARS UCD1.2.98). Subsequent analysis was performed in R (version 4.1.0). Gene counts based on the aligned reads were quantified using the FeatureCounts function of the Rsubread package^63^.

Principal component analysis was performed using the 500 most variable genes (based on variance of RPKMs across all samples). The limma package was used for differential expression analysis by following the authors workflow, with genes considered significantly differentially expressed above a log2-fold change cutoff of 1, and a false-discovery rate (FDR) below 0.05^64,65^.

### Flow cytometry

Flow cytometric analysis was performed on FAPs and SCs using PDGFRα-Alexa Fluor (AF) 647 (1:50; Abcam, ab270085), ITGA5-APC (1:50; Miltenyi Biotec, 130-110-591), CD14-APC (1:50; Miltenyi Biotec, 130-110-578), CD61-APC (1:50; Miltenyi Biotec, 130-110-887), combined with CD56-PE (1:10; BD Biosciences, 345812) antibodies on a MACSQuant10 flow cytometer (Miltenyi Biotec). Unstained cells were used routinely as negative controls and to define gating parameters. Flow cytometric data was analysed using FlowJo (FlowJo LLC, version 10.7.1).

### Immunofluorescence microscopy

Cells were fixed (4% formaldehyde, 10 min, RT) prior to analysis. For assessing 2D adipogenic differentiation, cells were stained with Hoechst 33342 (1:5000; Thermo Fisher, H3570) and BODIPY 493/503 (1:1000; Thermo Fisher, D3922; 30 min, RT). For myogenic differentiation, cells were permeabilised, blocked (5% BSA in PBS, 1 h, 4 °C), and stained with α-desmin antibody (1:100; Sigma-Aldrich, D1033; overnight, 4 °C), Hoechst 33342 and appropriate α-mouse secondary antibody (1:200; Thermo Fisher, A28175; 1 h, RT).

Immunofluorescence images were captured, and adipogenic and myogenic differentiation quantified, using an ImageXPress Pico High Content Analyser (Molecular Devices, LLC). Nuclei counts were determined by quantifying Hoechst staining, and BODIPY immunoreactivity was assigned to the nearest nuclei using MetaXPress software. BODIPY positivity was then determined by quantifying BODIPY positive nuclei as a proportion of total nuclei. Fusion index was calculated as the percentage of nuclei located within desmin-stained myotubes^41,42^. For metabolite spiking experiments, cells were fixed, stained with Hoechst, and quantified using MetaXPress.

### Adipogenic microfibre cell culture

FAPs were resuspended in 0.5% high viscosity non-functionalised alginate solution (Sigma-Aldrich, W201502) at a concentration of 3 × 10^7^ cells/mL. Cell/alginate suspension was injected into gelation buffer (66 mM CaCl_2_, 10 mM HEPES). The resultant microfibres were washed and transferred to 12-well tissue culture plates containing serum-free adipogenic differentiation medium (ADM; Supplementary Table 1). Medium without inducers served as control. Microfibres were incubated on a shaking platform (75 rpm, 37 °C, 5% CO_2_) and medium exchanges performed every 3–4 days.

### Immunofluorescence for adipogenic microfibres

Adipogenic microfibres were fixed in fixation buffer (4% formaldehyde, 66 mM CaCl_2_, 10 mM HEPES; 1 h, RT), and stained overnight at 4 °C (1:500 BODIPY 493/503, 1:625 Hoechst 34850). For immunocytochemistry, microfibres were permeabilised in blocking solution (66 mM CaCl_2_, 10 mM HEPES, 10% goat serum, 0.1% Triton X-100; 1 h) prior to staining with antibodies for acetyl-CoA carboxylase (ACC; 1:200; Cell Signalling, #3676) or perilipin-1 (PLIN1; 1:400; Abcam, ab61682; both overnight, 4 °C) and appropriate secondary antibodies (1:200, Thermo Fisher, A32754 or A11058) in blocking solution (2 h, RT).

Images were captured on a confocal microscope (TCS SP8, Leica Microsystems) using a 25×/1.00 objective lens and 5 µm Z-steps. Nuclei counts and lipid volumes were quantified using a custom python script (contact authors for details) for three separate images per sample, and the total quantified lipid volume divided by the number of nuclei to calculate lipid volume per cell.

### Microcarrier cell culture

Cytodex 1 microcarriers (GE Healthcare) were hydrated in PBS in siliconised (Sigmacote SL2, Sigma-Aldrich) 30 mL spinner flasks (Wheaton) at a final concentration of 10 cm^2^/mL. Prior to inoculation, microcarriers were preconditioned with GM (1 h, 37 °C, 5% CO_2_). Cells were seeded at 1.5 × 10^3^ cells/cm^2^. Spinner flasks were positioned on magnetic stirring platforms in an incubator, and agitated at 60 rpm. 50% medium exchanges were performed every 48 h. Brightfield and fluorescence microscopy images were captured with an EVOS M5000 microscope, and cell counts performed on an automated cell counter (NucleoCounter NC-200, Chemometec).

For cell harvesting, microcarriers were washed with PBS and incubated in 2X TrypLE (Gibco, A12177; 10 min, 37 °C, 200 rpm). Cells were separated from microcarriers using a vacuum driven 100 µm Steriflip filter unit (Merck Millipore, C3238) and several washing cycles. Cells were harvested by centrifugation (350 × *g*, 5 min), resuspended in GM and counted prior to subsequent handling or analysis.

### Scanning electron microscopy (SEM)

Samples were fixed in 2% formaldehyde, 2.5% glutaraldehyde (Sigma Aldrich, G5882) in 0.1 M cacodylate (Sigma Aldrich, C0250) for 24 h. Samples were rinsed with 0.1 M cacodylate, dehydrated in a graded ethanol series, and dried in hexamethyldisilazane (Sigma Aldrich, 440191). Samples were sputter-coated with a thin gold layer (SC7620 Mini Sputter Coater, Quorum Technologies) prior to observation on a scanning electron microscope (JSM-IT200, JEOL).

### Lipidomic analysis

10–30 mg samples were collected for lipidomic analysis. Lipids were extracted using a modified Bligh-Dyer protocol, and analysed by hydrophilic interaction liquid chromatography mass spectrometry (HILIC LC-MS/MS)^66^. Lipid quantities were normalised to the amount of protein present within the respective sample.

For data analysis, peak integration was performed with MultiQuantTM (version 3.0.3). Lipid species signals were corrected for isotopic contributions (calculated with Python Molmass 2019.1.1) and quantified based on internal standard signals as per the Lipidomics Standards Initiative (LSI).

### Sensory analysis

Sensory analysis was performed by three consenting, unblinded volunteers, who each regularly consume beef. Cultured fat was frozen and stored (−20 °C) prior to analysis. Frozen bovine subcutaneous fat and empty alginate hydrogel were provided as control samples. Samples were pan-fried (without oil) for 3–5 min, and samples scored numerically on a range of visual, olfactory and gustatory characteristics. All analyses were performed in accordance with EC Guidance on Ethics in Food Research^67^, and in compliance with Mosa Meat B.V. standard operating procedures. Ethical approval was waived under Dutch MREC/CCMO regulation.

### Statistical analysis

Statistical analysis was performed using Prism 9.1.0 (GraphPad). Analysis between two groups was performed using a Student’s *t*-test. Analysis of three or more groups was performed using one-way ANOVA with Bonferroni’s multiple comparisons test against indicated control(s) (Fig. 4d; Supplementary Figs. 1b; 5b; Supplementary Figs. 2b; 6c–f). Analysis of three or more groups with two independent variables was performed using a two-way ANOVA with Bonferroni’s multiple comparison test against indicated control(s) (Fig. 4b).

### Reporting summary

Further information on research design is available in the Nature Research Reporting Summary linked to this article.

## Supplementary information

Supplementary Material

## Data Availability

RNA sequencing data has been deposited online at the GEO, accession number GSE189296. Further data supporting the findings of this study are available from the authors upon request.

## Code availability

Code for analysis of 3D adipogenic differentiation images is available from the authors upon request (for academic use only).
