# Supplementary material for: Muscle-derived fibro-adipogenic progenitor cells for production of cultured bovine adipose tissue
Source: NPJ Sci Food. 2022 Jan 24;6:6. doi: 10.1038/s41538-021-00122-2 (PMC8786866; doi:10.1038/s41538-021-00122-2)
Supplement: Supplementary file 1 — Supplementary Material [file 41538_2021_122_MOESM1_ESM.pdf]

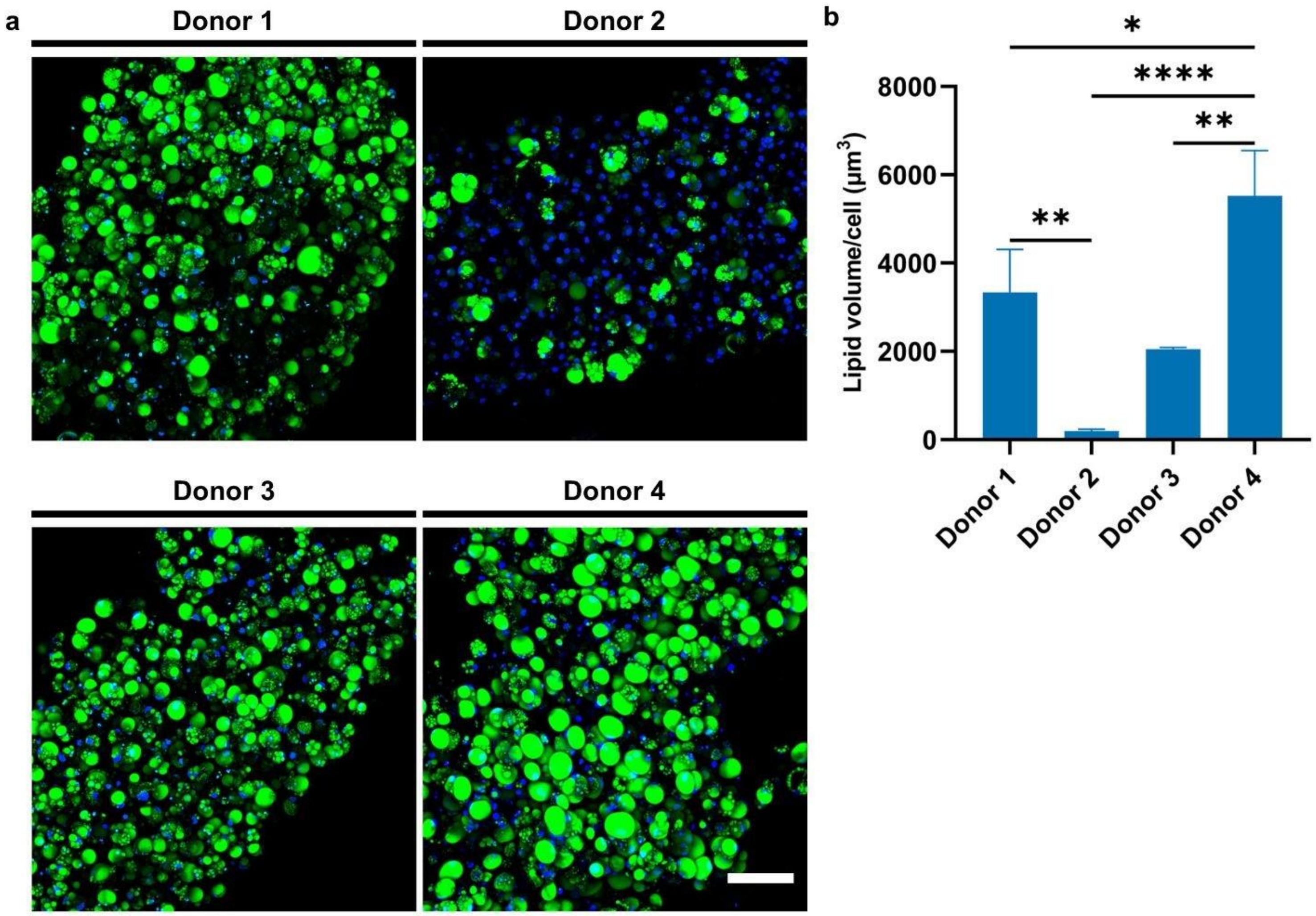

Supplementary Figure 1 | Adipogenic differentiation of FAPs in 3-dimensional hydrogels (related to Fig. 4).

a Maximum intensity projection confocal microscopy images of differentiated microfibres created with FAPs derived from four separate donor animals. Green = BODIPY, blue = Hoechst, scale bar = 100 µm.

b Lipid volume per cell, for samples in **a** (quantified by calculating total lipid volume from BODIPY immunofluorescence, divided by number of nuclei). Data is shown as mean ± sd (n = 3). *P*-values: *, $P \leq 0.05$; **, $P \leq 0.01$; ***, $P \leq 0.001$; ****, $P \leq 0.0001$.

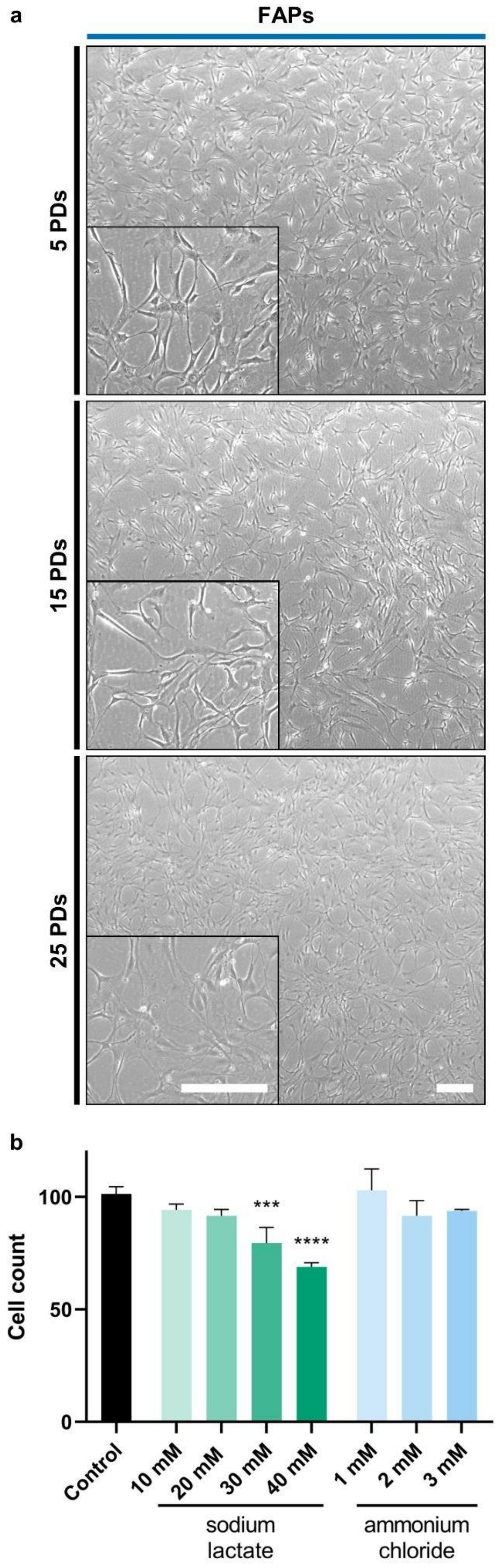

Supplementary Figure 2 | Proliferation of FAPs in long-term and upscaled cultures (related to Fig. 5).

a Brightfield microscopy images of proliferating FAPs at early, middle and late passage. Scale bars = 200 µm.

b Tolerance of FAPs to addition of waste metabolites, indicated by cell count after a 3 day culture. Counts were normalised and compared to control, and shown as mean ± sd (n = 3). *P*-values: *, $P \le 0.05$; **, $P \le 0.01$; ***, $P \le 0.001$; ****, $P \le 0.0001$.

Supplementary Table 1 | Media formulations

Component	Concentration
Growth medium (GM)	
Ham's F-10 Nutrient Mix (Gibco, 31550-023)	
Heat-inactivated Fetal Bovine Serum (FBS; Gibco, 10500064)	20%
Recombinant human bFGF (R&D systems, 233-FB)	5 ng/mL
Penicillin/Streptomycin/Amphotericin (PSA; Lonza, 17-745E)	1%
Myogenic differentiation medium	
DMEM (Gibco, 22320-022)	
Heat-inactivated FBS	2%
PSA	1%
Adipogenic differentiation medium (ADM), base	
DMEM (high glucose; Thermo Fisher, 41966-029)	
Chemically-defined FBS replacement	1%
PSA	1%
ADM - Inducers	
Human Insulin (Peprotech, 10-365)	10 µM
Rosiglitazone (Sigma-Aldrich, R2408)	5 µM
ADM - Inducers (first 3 days only)	
IBMX (Sigma-Aldrich, I5879)	0.5 mM
Dexamethasone (Sigma-Aldrich, D4902)	1 µM

Supplementary Table 2 | Primer sequences for RT-qPCR

Gene		Primer sequence
ADIPOQ	Fwd:	5’-GGCTCTGATTCCACACCTGA
	Rev:	5’-TGTTGTCCTCGCCATGACTG
B2M	Fwd:	5’-TGGAGGTGCTGGCATCTTAG
	Rev:	5’-ATGCAGAAGACACCCAGATGTT
CAV3	Fwd:	5’-GATCGATCTGGTGAACCGGG
	Rev:	5’-TGTAGCTCACCTTCCACACG
CIDEC	Fwd:	5’-TGCAGAGTAACCACTGCTGA
	Rev:	5’-ACGCCAGCATCAGGGTATC
DES	Fwd:	5’-GGAAGCCGAGGAATGGTACA
	Rev:	5’-TCGATCTCGCAGGTGTAGGA
FABP4	Fwd:	5’-GTAGGTACCTGGAAACTTGTCT
	Rev:	5’-ACTTTCCTGGTAGCAAAGCC
ITGB1 (CD29)	Fwd:	5’-TGAGGCCACTGTTCATGTTGT
	Rev:	5’-CCGTGTCCCATTTGGCATT
MYOG	Fwd:	5’-GCGCAGACTCAAGAAGGTGA
	Rev:	5’-TGCAGGCGCTCTATGTACTG
NCAM1 (CD56)	Fwd:	5’-CCGAGAAGGGTCCCGTAGA
	Rev:	5’-ATTTGTGTGGCATCGTTGGG
PAX7	Fwd:	5’-CTCCCTCTGAAGCGTAAGCA
	Rev:	5’-GGGTAGTGGGTCCTCTCGAA
PDGFRA	Fwd:	5’-TCTGCCAGCTTTCATTACCC
	Rev:	5’-TGACCACTTCCAGCACTGTC
RPLP0	Fwd:	5’-GGCAGCATCTACAACCCTGA
	Rev:	5’-CAGATGCGACGGTTGGGTAA
RPL19	Fwd:	5’-TCGAATGCCCGAGAAGGTAAC
	Rev:	5’-CTGTGATACATGTGGCGGTC
TNNT1	Fwd:	5’-CCTCTGATCCCGCCAAAGAT
	Rev:	5’-GGTCCTTTTCCATGCGCTTC
TRARG1	Fwd:	5’-CTCATCCTTGCCATCGCCTC
	Rev:	5’-TGTTGCACGCTACTTCGAGA
UXT	Fwd:	5’-GAGCAGTCTCCTCACAGAGCTC
	Rev:	5’-AGCAACATGTGGATATGGGCCT
